# Relapses of idiopathic inflammatory myopathies after vaccination against COVID-19: a real-life multicenter Italian study

**DOI:** 10.1007/s11739-022-03028-3

**Published:** 2022-06-26

**Authors:** Edoardo Conticini, Miriana d’Alessandro, Silvia Grazzini, Marco Fornaro, Daniele Sabella, Giuseppe Lopalco, Federico Giardina, Serena Colafrancesco, Chiara Rizzo, Giuliana Guggino, Roberta Priori, Fabrizio Conti, Florenzo Iannone, Elena Bargagli, Luca Cantarini, Bruno Frediani

**Affiliations:** 1 Rheumatology Unit, Department of Medical and Surgical Sciences and Neurosciences, University of Siena, 53100 Siena, Italy; 2 Respiratory Diseases Unit, Department of Medical and Surgical Sciences and Neurosciences, University of Siena, Viale Bracci 1, 53100 Siena, Italy; 3 Rheumatology Unit, Department of Emergency and Organ Transplantation, University of Bari, Policlinico, Piazza G. Cesare 11, 70124 Bari, Italy; 4 Rheumatology Section, Department of Clinical Internal, Anesthetic and Cardiovascular Sciences, Azienda Ospedaliero-Universitaria Policlinico Umberto I, Sapienza University of Rome, Rome, Italy; 5 Rheumatology Section, Department of Health Promotion, Mother and Child Care, Internal Medicine and Medical Specialties, University of Palermo, Piazza delle Cliniche 2, 90110 Palermo, Italy

**Keywords:** COVID-19 vaccination, Idiopathic inflammatory myopathies, Relapses

## Abstract

Severe acute respiratory syndrome coronavirus-2 (SARS-CoV-2) vaccination plays a crucial role as pivotal strategy to curb the coronavirus disease-19 (COVID-19) pandemic. The present study described the clinical status of patients affected by idiopathic inflammatory myopathies (IIM) after COVID-19 vaccination to assess the number of relapses. We included all patients affected by IIM and followed by Myositis Clinic, Rheumatology and Respiratory Diseases Units, Siena University Hospital, Bari University Hospital, Policlinico Umberto I, Sapienza University, Rome, and Policlinico Paolo Giaccone, Palermo. They underwent a telephone survey. A total of 119 IIM patients (median, IQR 58 (47–66) years; 32males; 50 dermatomyositis, 39 polymyositis and 30 anti-synthetase syndrome) were consecutively enrolled. Except four patients who refused the vaccination, 94 (81.7%) received Comirnaty, 16 (13.9%) Spikevax, 5 (4.4%) Vaxzevria. Seven (6.1%) patients had flare after vaccination. One of them had life-threatening systemic involvement and died two months after second dose of COVID-19 vaccination. From logistic regression analysis, Chi^2^-log ratio = 0.045,the variable that most influences the development of flare was the number of organs involved (*p* = 0.047). Sixty-eight patients received the third dose of COVID-19 vaccination: 51(75%) Comirnaty and 17 (25%) Moderna. No patients had flares after third dose. Our study represents the largest cohort of IIM patients in which the incidence of recurrence after anti-SARS-CoV-2 vaccine was assessed. In line with real-life data from other diseases, we found a clinical non-statistically significant risk of relapse in our patients, which occurred seldom, usually mild and in patients with a more severe and aggressive course of disease.

## Significance and innovation

- This is first of the studies to look at flares following COVID-19 vaccination longitudinally in idiopathic inflammatory myopathies multicentre cohort
- Clinical non-statistically significant risk of relapse in idiopathic inflammatory myopathies patients was found
- The risk of relapse after COVID-19 vaccination occurred seldom, and were usually mild and in patients with a more severe and aggressive course of disease.

## Introduction

Severe acute respiratory syndrome coronavirus 2 (SARS-CoV-2) vaccination plays a crucial role as pivotal strategy to curb the coronavirus disease-19 (COVID-19) pandemic. The use of recently developed mRNA vaccines, such as BNT162b2 (Pfizer) [1], and mRNA-1273 (Moderna) [2], and *ChAdOx1-S (AstraZeneca)* adenovirus vector vaccine [3], has provided effective protection against severe COVID-19. mRNA vaccines use lipid nanoparticles as a vehicle to deliver genetically modified mRNA. Once injected, the mRNA is translated into target protein resulting in robust immune response. To date, an excellent safety profile has been found for these vaccines [4] and severe reactions occur seldom.

Idiopathic inflammatory myopathies (IIM) constitute a heterogeneous group of myopathies characterized by immune-mediated inflammation of the striate muscle and an altered immune system [5]. In patients affected by such diseases, COVID-19 vaccination may cause an underlying inflammatory disease to flare or reduce immune responses.

Despite the mass-scale COVID-19 vaccination, literature data about the incidence of disease flares in IIM patients is not still reported as well as the immunological responses condition.

The present study aimed to describe the clinical status of patients affected by IIM after vaccination against COVID-19 to assess the number of relapses in a cohort of Italian patients with such disease.

## Methods

### Study population

We included all patients affected by IIM and followed by Myositis Clinic, Rheumatology and Respiratory Diseases Units, Siena University Hospital, Bari University Hospital, Policlinico Umberto I, Sapienza University, Rome, and Policlinico Paolo Giaccone, Palermo.

Inclusion criteria were a recent (< 3 months) clinical and serological assessment before the survey and the fulfillment of 2017 American College of Rheumatology/European League against Rheumatisms (ACR/EULAR) classification criteria for patients affected by Dermatomyositis (DM) and Polymyositis (PM) [6]; conversely, for patients affected by Anti-synthetase syndrome (ASS), due to the lack of validated criteria, inclusion criteria were positivity of any anti-synthetase antibody and a diagnosis performed by a physician with an expertise in the field of IIM.

Exclusion criteria were a diagnosis of inclusion body myositis (IBM), the non-completion of anti-SARS-CoV-2 vaccine cycle and the lack of a recent (< 3 months) assessment before survey.

All patients included in the study underwent a telephone survey to establish their clinical status and potential relapses after vaccination. When applicable, patients were evaluated in the outpatient clinic, as part of our clinical practice. When it was not possible (e.g. patient in full remission or living far from our centers, evaluated on a 6-month-based routine), patients’ data were collected by telephone interview.

The following data were collected: age, sex, definite diagnosis, antibodies, length of disease, number of organs involved, myositis damage index (MDI), physician global assessment (PhGA) in Likert scale, serum C-reactive protein (CRP), erythrocyte sedimentation rate (ESR), creatine kinase (CK), myoglobin and aldolase, treatment at the time of vaccination, including glucocorticoids (GCs) dosage, type of anti-SARS-CoV-2 vaccine, the onset of flare after vaccination, its type and severity (graded as mild, minor, major and life-threatening) and the change in medications after the flare. Flare was defined as worsening of MMT-8 by ≥ 20% or extra-muscular organ disease activity worsening by ≥ 2 cm on a 10-cm VAS or any of the IMACS CSMs worsening by ≥ 30% [7, 8] occurring within 30 days from vaccination against COVID-19.

All patients gave their written informed consent to participation in the study.

### Statistical analysis

We expressed all values as medians [interquartile ranges (IQRs)] or numbers (%). For categorical variables, we applied Fisher’s exact or Chi-squared tests to compare proportions between groups, and the Mann–Whitney U-test to compare medians. We applied logistic regression analysis to identify variables associated with flare after COVID-19 vaccination and the calculated odds ratio (OR), 95% confidence interval (CI), and P values. A p value less than 0.05 was considered statistically significant. Statistical analysis was performed by GraphPad Prism 9.3 and XLSTAT 2021 software.

## Results

A total of 115 IIM patients (median, IQR 58 (47–66) years; 30 males) were consecutively enrolled. Forty-eight had a diagnosis of DM, 37 had PM and 30 had ASS. The median months of disease duration was 79.62 ± 83.98. According to the extent of disease, thirty-six had only one organ or system involved, 45 had two, 22 had three, 11 had four and one had five.

The majority of them received two doses of COVID-19 vaccine, except four patients who refused the vaccination: 94 (81.7%) were vaccinated with Comirnaty (Pfizer BioNtech), 16 (13.9%) with Spikevax (Moderna), 5 (4.3%) with Vaxzevria (AstraZeneca). Seven (6.1%) patients had flare after vaccination, most of them were mild except one major with three organs involved and one life-threatening systemic involvement who died after two months from second dose of vaccination.

Table 1 shows demographic, clinical and immunological data in the two groups divided according to the development of flare after two doses of COVID-19 vaccination.

**Table 1:** Clinical, demographic and immunological features of IIM patients at the time of COVID-19 vaccination

Parameters	Flare after two doses (*n* = 7)	No-flare after two doses (*n* = 108)	*p* value
Age (years)	55 (51–68)	59 (47–67)	NS
Gender (male/female)	2/5	28/80	NS
*Diagnosis (n, %)*			NS
DM	2 (29%)	46 (43%)	
PM	2 (29%)	35 (32%)	
ASS	3 (42%)	27 (25%)	
*Antibodies (n, %)*			
Jo1	2 (29%)	25 (23%)	
PL7	–	3 (3%)	
PL12	–	1 (0.9%)	
Ku	–	2 (2%)	
Mi2	1 (14%)	7 (6.5%)	
PM/Scl	1 (14%)	5 (4.6%)	
Ro52	1 (14%)	7 (6.5%)	
TIG1g	–	5 (4.6%)	
MDA5	–	6 (5.5%)	
SRP	–	1 (0.9%)	
SAE	–	2 (2%)	
cN1a	–	–	
NPX	–	1 (0.9%)	
SSA	–	12 (11%)	
Ds-DNA	–	1 (0.9%)	
ANA (only positivity)	–	3 (3%)	
Negative	2 (29%)	27 (25%)	
Length of disease (months)	88.62 ± 105.02	78.35 ± 82.58	NS
*Number of organs involved (n, %)*			0.0004
One	0	36 (33%)	
Two	2 (29%)	43 (40%)	
Three	3 (43%)	19 (18%)	
Four	1 (14%)	10 (9%)	
Five	1 (14%)	0	
*Type of vaccination (n, %)*			NS
Comirnaty	6 (86%)	88 (81%)	
Spikevax	1 (14%)	15 (14%)	
Vaxzevria	0	5 (5%)	
*Disease activity (n, %)*			NS
PhGA ≥ 2	3 (43%)	27 (25%)	
PhGA < 2	4 (57%)	81 (75%)	
MDI	3 (1–6.5)	2 (1–4)	NS
CRP (mg/dL)	0.1 (0.01–0.3)	0.99 (0.3–2.9)	0.0041
ESR	32 (14–39)	15.5 (8–27.5)	NS
CPK	111 (63–905)	97.5 (63–158)	NS
Treatment at time of vaccination (*n*, %)			NS
GCs	0	10 (9%)	
Immunosuppressive	3 (43%)	19 (18%)	
Biologic	1 (14%)	2 (2%)	
Combination	3 (43%)	65 (60%)	
No-treatment	–	12 (11%)	

Fisher’s exact or Chi-squared tests were used to compare proportions of gender, diagnosis, organ involvement, type of vaccination, disease activity and treatment. Mann–Whitney *U* test analysis was used to compare medians

*ASS* anti-synthetase syndrome, *DM* dermatomyositis, *PM* polymyositis, *PhGA* physician global assessment, *MDI* myositis damage index, *CRP* C-reactive protein, *ESR* erythron with sedimentation, *CPK* creatin phosphokinase, *GCs* glucocorticoids

Clinical and demographic features of patients who had flare after second dose of vaccination against COVID-19 are reported in Table 2.

**Table 2:** Clinical and demographic data of IIM patients who had flare after second dose of vaccination against COVID-19, including age, gender type of vaccination, diagnosis, number of organs involved, treatment at the time of vaccination, severity of flare, type of flare, change in medication and outcome

Parameters	Patient 1	Patient 2	Patient 3	Patient 4	Patient 5	Patient 6	Patient 7
Age (years)	28	42	47	55	68	52	51
Gender (M/F)	F	F	F	F	F	M	M
Type of Vaccination	Moderna	Comirnaty	Comirnaty	Comirnaty	Comirnaty	Comirnaty	Comirnaty
Diagnosis	PM	ASS	ASS	DM	PM	PM	DM
Number of organs involved	Muscle	Skin and joint	Muscle	Muscle, skin, lung and GI	Muscle, skin, lung, heart and GI	Muscle and lung	Muscle and skin
Treatment at the time of vaccination	IVIG	GCs, MTX, RTX	MTX	GCs and MTX	MMF	RTX, Tacrolimus and GCs	GCs and MTX
Severity of flare	Mild	Mild	Major	Mild	Mild	Life-threatening	Mild
Type of flare	Muscle	Muscle	Muscle, heart and GI	Skin	Muscle	Macrophage activation syndrome	Muscle
Change in medication	No	Yes	Yes	Yes	No	Yes	No
Outcome	Resolved	Resolved	Resolved	Resolved	Resolved	Death	Resolved

To understand or predict the effect of demographic (gender, age) and clinical (number of organs involved, length of diseases, CPK values and disease activity) features on the flare development after vaccination, a logistic regression analysis was performed (Table 3).

**Table 3:** Results of logistic regression analysis for the development of flare after two doses of COVID-19 vaccination

	OR	95% CI	*p* value
Men (vs women)	0.66	0.04–10.83	0.770
Age (years)	0.91	0.79–1.06	0.227
Number of organs involved	5.77	1.03–32.44	0.047*
Length of diseases (months)	1.00	0.99–1.01	0.179
Disease activity (PhGA ≥ 2)	1.67	0.56–3.99	0.116
CPK	1.003	1–1.005	0.061

^*^p < 0.05

**Fig. 1:**
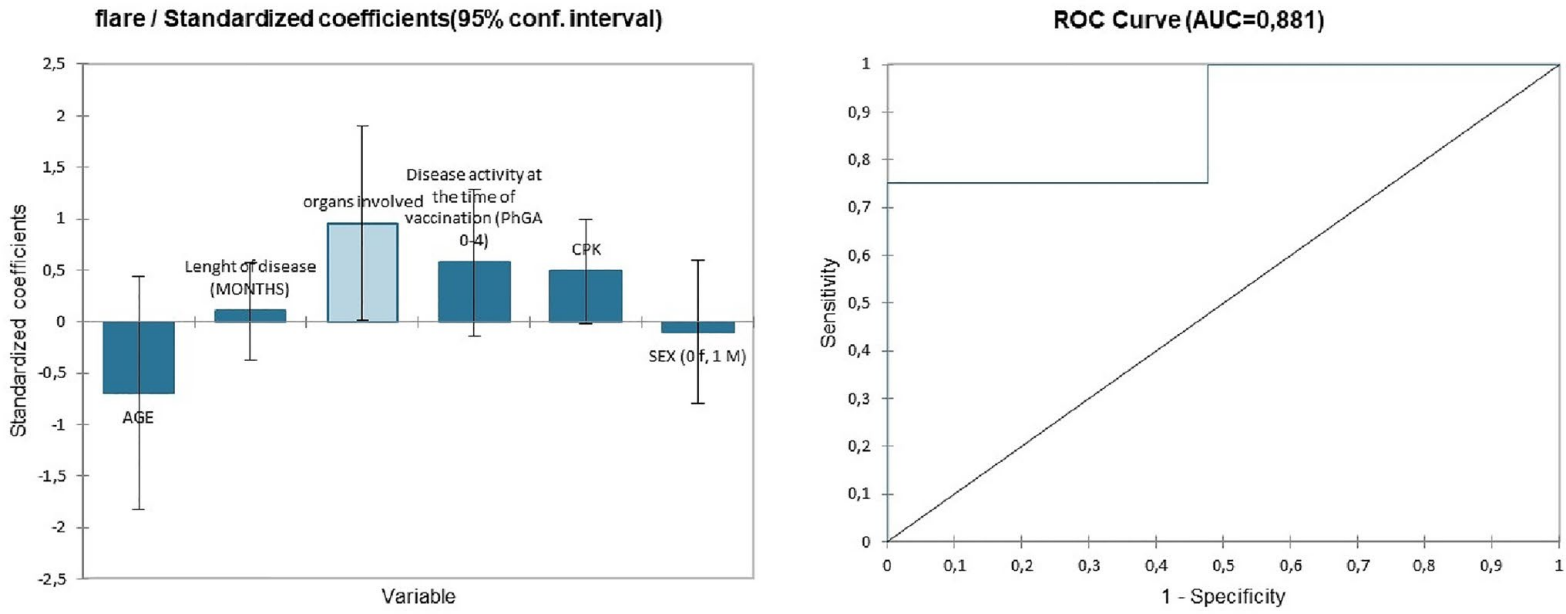
**a**, the left. Logistic regression analysis to predict the effect of demographic (gender, age) and clinical (number of organs involved, length of diseases, CPK values and disease activity) features on the flare development after vaccination. The Chi^2^ associated with the Log ratio was 0.045. **b**, the right The ROC curve of the logistic regression model showed an AUC of 0.881

**Fig. 2:**
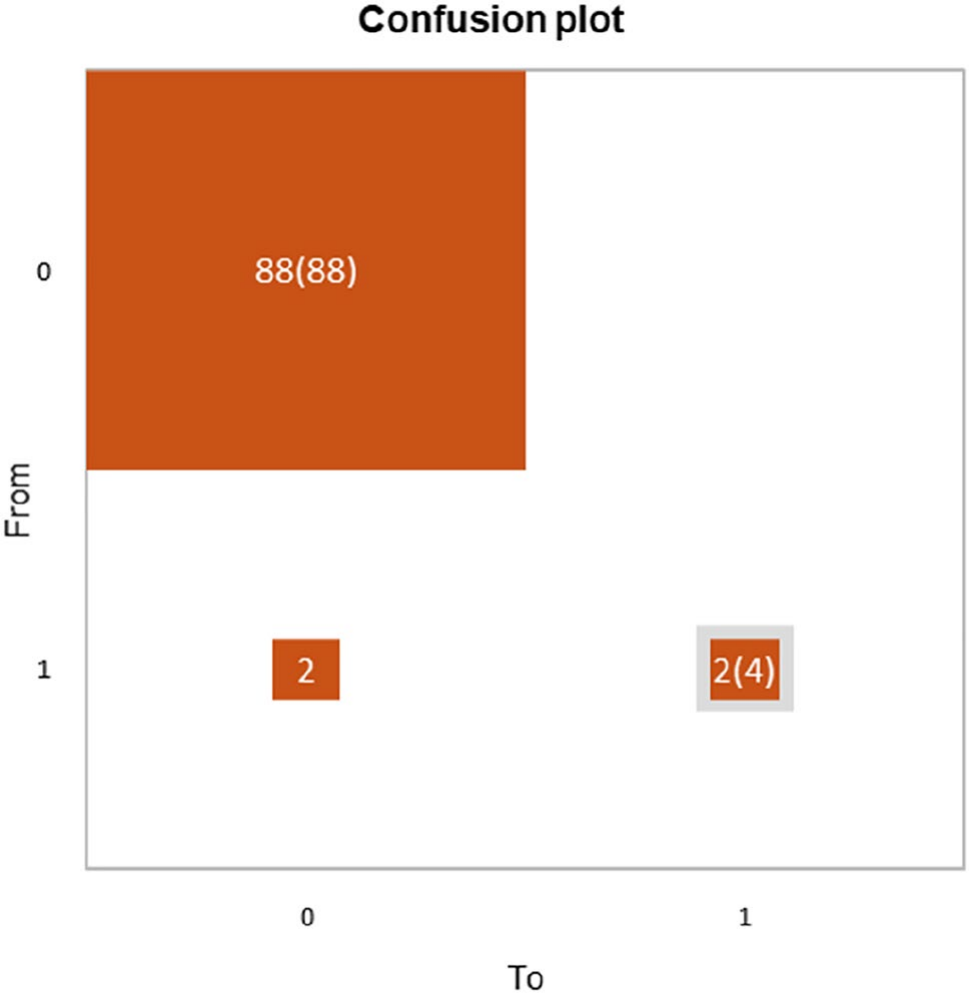
Confusion plot analysis based on the classification table for the training sample (confusion matrix) according to the flare after two doses of COVID-19 vaccination (0 = no flare, 1 = flare). The gray squares on the diagonal represent the observed numbers for each modality. The orange squares represent the predicted numbers for each modality

The goodness-of-fit statistics showed a Chi^2^ associated with the Log ratio (L.R.) of 0.045 (Fig. 1a). From the probability associated with the Chi-square tests, the Type II analysis showed the variable that most influences the development of flare was the number of organs involved (*p* = 0.047). The ROC curve of the logistic regression model showed an AUC of 0.881 (Fig. 1b). Accordingly, the classification table for the training sample (confusion matrix) and confusion plot was performed (Fig. 2). The control group (patients w/o flare after vaccination) was well classified at 100% while patients who developed flare after vaccination were well classified at 50%.

Sixty-eight patients received the third dose of COVID-19 vaccination: 51 (75%) Comirnaty and 17 (25%) Spikevax. No patients had flares after the third dose of vaccination against COVID-19.

## Discussion

The approval of several vaccines against SARS-CoV-2 has dramatically changed perspectives of the global struggle against COVID-19. Such vaccines have provided, both in registrative studies [9] and in preliminary real-life evidence [10, 11], an overall good efficacy and safety.

Statistically significant reduction of death and hospitalization emerged in subjects completed 3-dose vaccination courses to those non-vaccinated [12]. Analogously, no relevant adverse events have been generally reported. Although registrative studies related to COVID-19 vaccination did not include patients affected by severe autoimmune diseases, after roughly one year after the start of the global vaccination campaign, a growing number of real-life data is available about efficacy and safety of anti-SARS-CoV-2 vaccines in patients affected by severe rheumatic disorders [13–15].

The very first evidence came from case reports or small case series, reporting the onset of autoimmune inflammatory disorders in previously healthy subjects or their relapse in patients considered in remission or in low disease activity [16].

The most common rheumatic adverse events in healthy subjects, aside from arthralgias, are seronegative arthritis [17], polymyalgia rheumatica [17, 18] and skin, urticarial or leukocytoclastic, vasculitis [19–21]. Specifically focusing on IIM, only scanty data are available: 5 papers, for a sum of 8 patients [22–26] mentioned the occurrence of myositis after vaccination and in all of them the course of disease was favorable, with an overall good response to treatment. Notably, in 3 of them a concomitant anti-SAE or anti-Pm/Scl 75 positivity was assessed.

Nevertheless, vaccines seem to be a safe option in rheumatic patients, as large studies, performed in wide cohorts, have not shown any significant risk of relapse in this particular subset of patients [27–30], even when affected by rare diseases [31, 32].

In line with these findings, there is a paucity of data for patients with a previous diagnosis of IIM who subsequently underwent anti-SARS-CoV-2 vaccine. To the best of our knowledge, roughly 100 myositis patients, coming from multicenter studies [14, 27, 28, 32–35], were included in studies focusing on vaccine safety, while other ones, although larger, did not include IIM [29, 36]. Moreover, only two papers specifically assessed the risk of flares among these patients [27, 28], while none of them stratified patients according to disease activity, number of organs involvement, current and previous treatments nor assessed risk of flares after booster doses.

Efficacy of anti-SARS-CoV-2 vaccines in specific categories of patients, due to the lack of validated diagnostic procedures, has been even less assessed; nevertheless, a good protection from severe forms of COVID-19 seems to be provided also in patients affected by rheumatic diseases [37] in general and IIM in particular [38], despite a lower rate in seroconversion in subjects treated with anti-CD20 and anti-CTLA-4 agents [39, 40].

To the best of our knowledge, ours represent the largest cohort of IIM patients in which the incidence of recurrence after anti-SARS-CoV-2 vaccine was assessed. Moreover, in our study, we specifically assessed disease extent, activity and damage, as well as the autoimmune profile and the concomitant immunosuppressive treatment, to assess which patients were more prone to suffer from disease relapse. Finally, ours is the first study to assess the incidence of recurrence after the “booster” dose of anti-SARS-CoV-2 vaccine, which has now gained a paramount role in the protection against “Omicron” variant [10].

In our cohort, only a minority of patients (7 out of 115) suffered from any relapse after the first two doses of vaccine and only one of them had a major flare of disease. Similarly, an even lower incidence of flare was evidenced in those patients who underwent “booster” dose of vaccine: a further disease relapse was assessed only in the one who suffered from a major flare after the second dose.

Stratifying our patients according to the flare after COVID-19 vaccination, the number of organs involved, and CRP values were statistically different. Assessing logistic regression analysis only the number of organs and systems could affect flare after COVID-19 vaccination: that means that patients with a more severe and aggressive disease, namely the ones with extra-muscular involvement, may be more prone to suffer from IIM flare, presumably due to the incidence of recurrence after COVID-19 vaccination burden and to a less controlled disease.

On the other hand, such patients are the ones who, due to the overall systemic involvement, namely the respiratory tract one, and the prolonged immunosuppression status, have the worst outcome in case of COVID-19 pneumonia [41]: for this reason, also in this subset of patients, anti-SARS-CoV-2 vaccination should be strongly suggested nor delayed.

Despite the contribution of our study to evaluate the safety of vaccination against COVID-19 in IIM patients, we did not analyze the rate of recurrence after COVID-19. Further studies should be performed to assess the occurrence of myositis relapses in COVID-19 and their optimal management.

In conclusion, we evidenced a good safety profile of anti-SARS-CoV-2 vaccine in a large cohort of patients affected by IIM. Our findings, which are in line with real-life data coming from patients with other diseases, have found a clinical non-statistically significant risk of relapse in our patients, which occurred seldom, usually mild and in the ones with a more severe and aggressive course of disease: indeed, the only patient who died suffered from a severe, long-standing PM poorly responsive to the treatment Such findings are comparable with the literature data available about other rheumatologic disorders [30, 42].

## Data Availability

The data presented in this study are available on request from the corresponding author.

## Abbreviations

COVID-19: Coronavirus disease-19
IIM: Idiopathic inflammatory disease
SARS-CoV-2: Severe acute respiratory syndrome coronavirus 2
